# Long-term culture of mesenchymal stem cells impairs ATM-dependent recognition of DNA breaks and increases genetic instability

**DOI:** 10.1186/s13287-019-1334-6

**Published:** 2019-07-29

**Authors:** Daniela Hladik, Ines Höfig, Ursula Oestreicher, Johannes Beckers, Martina Matjanovski, Xuanwen Bao, Harry Scherthan, Michael J. Atkinson, Michael Rosemann

**Affiliations:** 1 Institute of Radiation Biology, Helmholtz Zentrum München GmbH, 85764 Neuherberg, Germany; 2 BfS Federal Office for Radiation Protection, Ingolstaedter Landstr. 1, 85764 Neuherberg, Germany; 3 Institute of Experimental Genetics, Helmholtz Zentrum München GmbH, 85764 Neuherberg, Germany; 4 German Center for Diabetes Research (DZD), 85764 Neuherberg, Germany; 5 Chair of Experimental Genetics, Technische Universität München, Wissenschaftszentrum Weihenstephan, 85354 Freising, Germany; 6 Bundeswehr Institute of Radiobiology, University of Ulm, Neuherbergstr. 11, 80937 Munich, Germany; 7 Chair of Radiation Biology, Technical University of Munich, 81675 Munich, Germany; 8 Present Address: BioNTech IMFS, Vollmersbachstr. 66, 55743 Idar-Oberstein, Germany

**Keywords:** Ionizing radiation, Adult stem cells, Mesenchymal stem cells, Genetic instability, DNA repair, Micronuclei, In vitro aging

## Abstract

**Background:**

Mesenchymal stem cells (MSCs) are attracting increasing interest for cell-based therapies, making use of both their immuno-modulating and regenerative potential. For such therapeutic applications, a massive in vitro expansion of donor cells is usually necessary to furnish sufficient material for transplantation. It is not established to what extent the long-term genomic stability and potency of MSCs can be compromised as a result of this rapid ex vivo expansion. In this study, we investigated the DNA damage response and chromosomal stability (indicated by micronuclei induction) after sub-lethal doses of gamma irradiation in murine MSCs at different stages of their in vitro expansion.

**Methods:**

Bone-marrow-derived tri-potent MSCs were explanted from 3-month-old female FVB/N mice and expanded in vitro for up to 12 weeks. DNA damage response and repair kinetics after gamma irradiation were quantified by the induction of γH2AX/53BP1 DSB repair foci. Micronuclei were counted in post-mitotic, binucleated cells using an automated image analyzer Metafer4. Involvement of DNA damage response pathways was tested using chemical ATM and DNA-PK inhibitors.

**Results:**

Murine bone-marrow-derived MSCs in long-term expansion culture gradually lose their ability to recognize endogenous and radiation-induced DNA double-strand breaks. This impaired DNA damage response, indicated by a decrease in the number of γH2AX/53BP1 DSB repair foci, was associated with reduced ATM dependency of foci formation, a slower DNA repair kinetics, and an increased number of residual DNA double-strand breaks 7 h post irradiation. In parallel with this impaired efficiency of DNA break recognition and repair in older MSCs, chromosomal instability after mitosis increased significantly as shown by a higher number of micronuclei, both spontaneously and induced by γ-irradiation. Multifactorial regression analysis demonstrates that in vitro aging reduced DNA damage recognition in MSCs after irradiation by a multiplicative interaction with dose (*p* < 0.0001), whereas the increased frequency of micronuclei was caused by an additive interaction between in vitro aging and radiation dose.

**Conclusion:**

The detrimental impact of long-term in vitro expansion on DNA damage response of MSCs warrants a regular monitoring of this process during the ex vivo growth of these cells to improve therapeutic safety and efficiency.

**Electronic supplementary material:**

The online version of this article (10.1186/s13287-019-1334-6) contains supplementary material, which is available to authorized users.

## Background

Tissue-resident mesenchymal stem cells (MSCs) of the adult organisms exhibit long-lasting self-renewing capacity and a multi-lineage differentiation potential [1, 2]. They contribute to the replacement of lost, damaged, or dysfunctional cells in mesenchymal tissues and therefore play a central role in wound healing, cellular homeostasis, and regeneration. Their capacities to migrate towards sites of inflammation and injury and to generate committed precursor cells are essential for this function [3]. Due to these unique properties, MSCs hold great promise as therapeutic vehicles in cell-based therapies [4]. Their self-renewal capacity and the ability to differentiate into various cell types of the mesenchymal lineage facilitated the development of applications in regenerative medicine and tissue repair. Clinical trials confirmed the therapeutic benefit of MSC-based therapies for bone healing [5], recovery of damaged cartilage [6, 7], and repair of large skin lesions [8]. In response to appropriate physiological signals, MSCs can divide in an asymmetric fashion giving rise to committed precursor cells for connective tissue, bone, cartilage, and adipose and even neuronal tissue [3, 5, 9, 10]. In recent studies, MSCs have also been shown to be beneficial for chronic cutaneous lesions after a high dose of irradiation to the skin [11]. In addition to their capacity to produce new precursor cells for tissue regeneration, MSCs also contribute to tissue repair by secreting anti-inflammatory cytokines [12]. It has been shown that MSCs can reduce immune reaction against HLA antigens, as for instance during graft-versus-host disease (GvHD) following bone marrow transplantation, where co-transplantation of MSCs can significantly reduce the clinical symptoms [13]. The homing capacity of MSCs to sites of inflammation is another mechanism with therapeutic potential [14], exploiting them as vectors for gene delivery in cancer therapy [15].

For the most part, the production of such transiently amplifying daughter cells with restricted differentiation potential does not interfere with the self-renewal capacity or the multi-potency of the second daughter cell. However, both MSC self-renewal and the generation of committed precursor cells require protection of the genome of the daughter cells from genomic instability.

MSCs can be harvested relatively easily from the bone marrow or adipose tissue of patients (for autologous transplantation) or of allogenic donors. Other less invasive tissue sources, for instance from dental pulp, hair follicles, or skin biopsies, yield a much smaller amount of starting material. This requires a more extensive in vitro expansion prior to transplantation. So far, it is not fully established to what extent MSCs retain their normal physiological function after transplantation, and whether they are able to properly graft and generate lineage committed precursor cells of the required differentiation potential [16]. Any form of cellular stress, which may impair potency and stability of MSCs during long-term culture should be minimized and carefully monitored in order to retain efficiency and safety of MSC-based therapies. One common form of genotoxic stress to MSCs results from ionizing radiation, either during diagnostic or therapeutic procedures to a patient or from common environmental sources. A single computer tomography investigation can lead to several 10 of mGy, i.e., the lowest dose that we have used in the current study. Natural background radiation is a completely unavoidable source of exposure, and its chronic nature could lead to a long-term accumulation of cellular alterations in stem cells with their slow turnover.

The proliferation capacity of MSCs in vitro remains a limiting factor despite optimized protocols, administration of defined growth factors, and reduction of growth-inhibitory conditions like oxidative stress. After a finite number of divisions in vitro, MSCs enter a state of senescence, characterized by reduced proliferation rate and changes in metabolic pathways [17]. Senescent MSCs lose their multipotent differentiation capacity which is associated with alterations in their epigenetic profiles, in particular changes in CpG methylation patterns [18]. This accelerated aging process in vitro is assumed to be caused by the absence of the protective environment of the stem cell niche in vivo*.* Studies using murine MSCs have shown that long-term in vitro growth can lead to karyotypic changes and an increase in the frequency of cells at risk of malignant transformation [19, 20]. Human MSCs can also exhibit clonal karyotypic alterations after long-term ex vivo expansion [21, 22]. It is not clear though whether this is associated with a malignant transformation [23], as can be induced for instance by genotoxic γ-radiation [24] or chemical carcinogens [25].

Human MSCs have a high survival capacity after ionizing radiation, as indicated by the resistance of the hosts’ hematopoietic stroma following high-dose therapeutic bone marrow irradiation [26]. In vitro tests for their colony formation capacity confirmed that human MSCs are relatively resistant for γ-rays or heavy ion irradiation [27]. Studies on the extent of DNA damage response on the level of cell-cycle checkpoints, DNA repair, and the signaling pathways involved (such as phospho-ATM, DNA-PK, and γH2AX) supported the concept that MSCs have a robust cellular radiation response [28]. Further evidence was found in a direct comparison showing that MSCs have a higher DNA repair capacity and better survival ratio after irradiation than more differentiated osteoblasts [29]. There is little evidence for an increased apoptosis in irradiated human or murine MSCs in vitro [30, 31]. The accumulating findings of a relatively high radioresistance suggest that MSCs will be able to maintain their proliferative potential even after high levels of radiation damage or other sources of genotoxic stress [30]. While cellular radio-resistance would increase the long-term regenerative capacity of MSCs after genotoxic stress, an unwanted consequence could be a higher rate of accumulated somatic mutations, when cells affected by genetic instability continue to proliferate. A high repair fidelity and an instant and potent DNA damage response (DDR) are therefore crucial for MSCs to avoid passing on mutations or other forms of genomic instability to their daughter cells.

The aim of this study was to investigate changes in DNA repair efficiency and genomic stability of murine MSCs during long-time ex vivo expansion. We used cells from genetically defined inbred FVB/N mice, which came from a very well controlled animal facility. In contrast to studies using human donor-derived MSCs, this reduced the possible influence of genetic heterogeneity and of variations coming from different health or physiological status to a minimum. Based on the current literature, we hypothesized that epigenetic alterations that occur during massive ex vivo expansion not only increase the rate of cell senescence, but could also render MSCs less responsive to genotoxic stress. We could show that the capacity of MSCs to recognize DNA double-strand breaks in the form of γH2AX/53BP1 repair foci is gradually lost when the cells continue to grow in vitro for up to 8 weeks. This goes in parallel with slower repair kinetics and an increase of spontaneous and radiation-induced micronuclei.

## Methods

### Cell culture of primary murine MSCs

Primary mesenchymal stromal cells were harvested from the femurs and tibias of female FVB/N mice (Charles River Laboratories, Sulzbach, Germany) maintained in a SPF breeding colony at the Helmholtz Centre, Munich. Following ethically approved asphyxiation of mice under flow-controlled CO_2_ gassing, the hind legs were dissected under sterile conditions and the entire bone marrow aspirated with 2 ml ice cold PBS and a 0.4-mm injection needle. Solid aggregates in the collected bone marrow suspension were separated by passing the suspension several times through a 1-ml pipette tip. Following centrifugation for 5 min at 300*g*, the resulting cell pellet was resuspended in 12 ml of growth medium (DMEM/HamF12 with 1 g/l glucose and GLUTAMAX) containing 10% MSC-approved FBS (Invitrogen, Thermo Fisher) and 10 μM rock-inhibitor Y-27632 (Tocris, Wiesbaden, Germany). The cell suspension was incubated under hypoxic conditions (2% O_2_, 5% CO_2_) at 37 °C in a humidified atmosphere. Non-adhering cells were depleted by medium exchange 2 and 4 h after initial plating, whereas later on, the medium was replaced every 3.5 days. When reached approximately 80% confluency, they were passaged in a 1:3 ratio once per week using StemPro accutase (Gibco). All experiments were done on at least 3 biological replicates (i.e., MSCs established from 3 mice). Representative pictures of MSC growing for 1 to 12 weeks are shown in Additional file 1: Figure S3. Three-lineage differentiation potential towards osteoblastic, chondroblastic, and adipogenic cells was regularly tested by lineage-specific inducers (Invitrogen, Thermo Fisher) and histochemical staining as recommended by the manufacturer (Additional file 2: Figure S4).

### γ-Irradiation of cells

In vitro γ-irradiation was performed in a closed cabinet Caesium-137 source (HWM-D-2000) at a dose rate of 460 mGy/min. For radiation doses below 0.5 Gy, a shielded lead box was used that reduced the dose rate by a factor of 10. Cells were kept at ambient temperature during irradiation, and control cells were treated identically apart from not placing them inside the radiation source.

### Immunofluorescence staining for DNA-repair foci

Cells were plated on tissue culture chamber slides (Ibidi, Martinsried, Germany) and on the following day irradiated with γ-doses between 0.05 and 6 Gy. To test for ATM and DNA-PK dependency, the chemical inhibitors KU55933 and NU7441 (KuDOS Pharmaceuticals, Cambridge, UK) were added to the cells 30 min prior to irradiation to a final concentration of 10 μM each. After the indicated repair incubation, cells were fixed for 10 min using Roti HistoFix (Carl Roth, Germany), washed twice with PBS, and permeabilized with 0.2% Triton X100 in PBS. After blocking for 1 h with 1% BSA, 0.15% glycine in PBS, samples were incubated with a combination of a mouse monoclonal anti-γH2AX antibody (Merck Millipore, Schwalbach, 1:500) and a rabbit polyclonal anti-53BP1 antibody (Novus Biological, Littleton, USA, 1:500) at 4 °C overnight. After 3 × 15 min washing in PBS/1% BSA, secondary antibodies (1,500 Cy3-conjugated sheep anti-mouse IgG and 1:200 Alexa 488-conjugated goat anti-rabbit IgG, Jackson Immunoresearch, West Grove) were diluted in PBS/1% BSA and applied for 1 h at room temperature to the slides. After a final washing step (3 × 15 min in PBS/1% BSA), slides were covered with Vectashield plus DAPI (VectorLabs, Burlingame, Ca) and mounted with glass coverslips.

Co-localized γH2AX and 53BP1 foci were detected using an electronic all-in-one fluorescent microscope (model BZ-9000; Keyence, Osaka, Japan) (Additional file 6: Figure S1), for which the 100× oil immersion lens and excitation and emission filter sets were optimized for Cy3, Alexa488, and DAPI. Using the Keyence BZ-II Analyser software, the recorded images (in 16bit tiff format) were processed for haze reduction and black balance correction, before sub-nuclear spots containing at least 70 pixels in the Cy3 and Alexa488 channel were automatically detected and counted as repair foci. At least 50 nuclei were analyzed for each experiment. To visualize the dose-response of radiation-induced DNA DSBs, the mean number of foci and the standard error of mean of counted foci were plotted vs. the radiation dose. For the analysis of the repair kinetics, the natural logarithm of DNA foci (mean number and standard error of mean after 2 Gy) was plotted vs. repair time and normalized to the number of foci counted at 90 min post-irradiation. The *t*_1/2_ of repair was calculated by best fitting the experimental data to an exponential decay curve.

### Micronucleus assay

For analysis of micronuclei (MN), 60,000 MSCs per 250-μl growth medium were seeded on sterile glass slides in an area of 1 cm × 2 cm. After the cells attached to the glass surface within 2 h, the slides were submerged in a 3-ml growth medium and incubated for another 48 h in the growth medium. Slides were irradiated with 0, 50 mGy, 500 mGy, 2 Gy, and 6 Gy γ-rays, and cytochalasin B was added to a final concentration of 4 μg/ml (to block cytokinesis). After 4 days of incubation at standard conditions, cells were fixed, rinsed with PBS, and stained with DAPI (Vectashield plus, VectorLabs, Burlingame, Ca).

For visualization and image acquisition of the cells, a Zeiss Axio Imager Z2 microscope mounted to a monochrome megapixel charge-coupled device camera was used. Optics consisted of a Xenon-UV excitation bulb, a × 10 lens, and filter sets for DAPI fluorescence. The image analysis software Metafer4 (MetaSystems, Altlussheim, Germany) was trained on murine MSCs to automatically scan an entire slide and find the bi-nucleated (BN) cells, based on their size, shape, and distance of the sister nuclei. False-positive BN cells were identified by eye and excluded from the analysis. The stored images of all BN cells from each slides were scored by eye for the presence of micronuclei (MN), yielding frequency distributions of BN cells with 0, 1, 2…10 micronuclei, mean number of micronuclei per BN cell, all based on a minimum of 150 BN cells per sample.

### Pulsed-field electrophoresis

MSCs growing in vitro for 1 and 8 weeks, resp., were γ-irradiated in cell culture dishes with doses between 1 and 50 Gy, incubated for 90 min at 37 °C, and collected using a cell scraper. After washing cells twice with ice-cold PBS and spinning down for 5 min at 300*g*, the resulting cell pellet was re-suspended in PBS (at 2 × 10^6^ cells/ml), mixed with an equal volume of 1% low melting agarose at 37 °C, and poured in volumes of 100 μl into the wells of a BioRad CHEF-DR2 gel plug mold. After solidifying for 15 min at 4 °C, agarose plugs were placed in a lysis solution containing 2% Sarkosyl, 1 mg/ml Proteinase K, and 500 mM EDTA for 2 h on ice and then for 16 h at 37 °C. After two washing steps with PBS, plugs were treated with 0.1 mg/ml RNaseA for 2 h at 20 °C. The plugs were then inserted into the wells of a 0.7% agarose gel and sealed with a thin layer of agarose. Pulsed-field gel electrophoresis was performed on a CHEF DR2 system (BioRad, Hercules, CA) in 0.5× TBE buffer at 14 °C with the following parameters: *T*_Pulse_ = 75 min, *T*_run_ = 24 h, *E* = 3 V/cm. The DNA in the gel was then stained for 12 h in TBE buffer containing 400 μg/l ethidium bromide, followed by a washing step in clear TBE buffer for 4 h. Gel images were visualized and stored using the electronic fluorescence gel documentation system AlphaImager 2200 (Alpha Innotec, Santa Clara CA) with UV transmission and camera filter sets for EtBr fluorescence. The 16-bit Tiff images of the gel were imported in ScionImage4.0.2 software (Scion Corp, NIH, USA) for densitometry analysis. The integrated pixel density of the fluorescent signals inside and outside of the well was quantified (after background correction), and the percentage of signal outside-the-well representing DNA fragmented by DSBs was plotted in linear mode vs. the irradiation dose.

### Statistical analysis

All data were analyzed using Statistica 13 (Dell Inc., Round Rock, TX). Results of multifactorial ANOVA are given with their respective *F* value, degrees of freedom, and *p* value. Multifactorial regression analysis was used to quantify the effects of in vitro aging, of the γ-radiation dose and their interaction. Results are expressed as linear regression coefficients *b*, *F* values, and *p* values. *T* test was done two-sided and paired (for the biological replicates) and results expressed as the *p* values. Chi^2^ test was done to compare observed micronuclei distribution with the expected values of a Poisson distribution and expressed as Chi^2^ values and *p* values.

## Results

### Induction of DNA repair foci is impaired in in vitro expanded MSCs

**Fig. 1:**
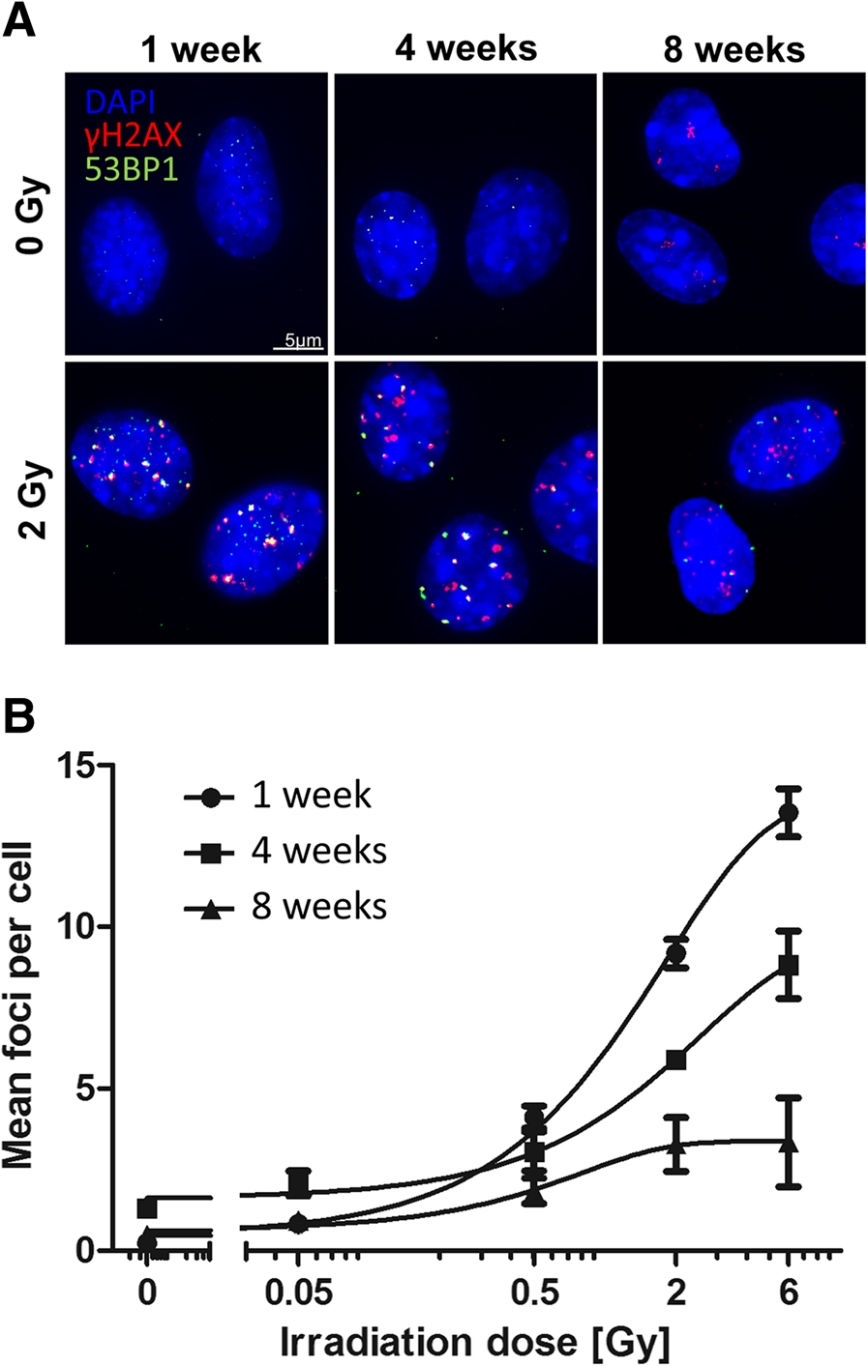
Induction of γH2AX- and 53BP1-foci formation after exposure to irradiation in aging MSCs in vitro. **a** MSCs were cultured for 1, 4, and 8 weeks in vitro. Repair foci formation is shown in MSCs 90 min after sham-irradiation (0 Gy) or after 2 Gy of γ-irradiation by immunofluorescence staining for γH2AX (red) and 53BP1 (green). Nuclear counterstaining was done using DAPI. **b** Quantification γH2AX- and 53BP1-foci formation in MSCs was done 90 min after sham-irradiation (0 Gy) or after γ-irradiation with 0.05, 0.5, 2, and 6 Gy using the Keyence BZ-II Analyzer software (see Additional file 6: Figure S1). Mean number of foci ± SEM (*n* = 3) are calculated from at least 50 analyzed nuclei

Primary mouse MSCs were expanded in vitro for 1, 4, and 8 weeks and tested for the induction of γH2AX and 53BP1 labeled repair foci of DNA double-strand breaks (DSB) following γ-radiation (Fig. 1a). Nuclear foci of co-localized γH2AX and 53BP1 signals occurred with a peak induction at around 90 min post-radiation with doses of 50 mGy, 500 mGy, 2 Gy, and 6 Gy. There was a dose response for the number of DNA-repair foci in cells at all ages. The youngest MSCs (1 week in culture) showed a significant increase of foci number after the lowest exposure of 50 mGy (*p* = 0.041). In 4- and 8-week-old MSCs, a significant increase of foci number above background occurred only at doses of 500 mGy and above (*p* = 0.047 and *p* = 0.026, resp.). In both cases, however, a steady rise in foci number was seen up to 6 Gy, with 1-week-old cells developing on average of 13.5 ± 0.7 foci/cell, compared with only 8.8 ± 1.0 foci/cell in 4-week and 3.3 ± 1.0 foci/cell in 8-week-old cells. The frequency of γH2AX/53BP1 foci in unirradiated cells, indicating spontaneous DNA DSBs, was not significantly different after 1-, 4-, and 8-week expansion in vitro (Fig. 1b). ANOVA test with radiation-dose and age in vitro as independent variables confirmed that the number of DSB foci exhibit a clear dose dependence in MSCs of all ages (Table 1) even though the increase reached significance in this pooled analysis only for doses of 500 mGy and higher. Multiple linear regression for the effects of both factors confirmed a dose-dependent increase of DSB foci number (*p* < 0.0001), a strong multiplicative interaction (both *p* < 0.0001) of dose (positively correlated) and age in vitro (negatively correlated), but no effect of age in vitro on its own (Table 1).

**Table 1:** Multiple linear regression for induced DNA DSB repair foci in MSCs indicates a strong multiplicative interaction (*F* = 4.8, *p* < 0.0001) between dose (positive effect with a coefficient of *b* = 2.26) and in vitro age (negative effect with a coefficient of *b* = − 0.24). In vitro age alone has no significant effect on the formation of DNA DSB foci

	Foci/cell	Foci/cell	Foci/cell	Foci/cell	− 95%	95%	Foci/cell	Foci/cell	− 95%	95%
	Param.	St.Err.	*T*	*p*	Conf.Int.	Conf.Int.	Beta	St.Err. Beta	Conf.Int.	Conf.Int.
Const	*2.299*	*0.524*	*4.388*	*0.0001*	*1.242*	*3.358*				
Dose	*2.265*	*0.185*	*12.263*	*0.000*	*1.892*	*2.638*	*1.343*	*0.109*	*1.122*	*1.564*
Age	− 0.112	0.101	− 1.107	0.2757	− 0.315	0.092	− 0.084	0.076	− 0.237	0.069
Dose × age	*− 0.238*	*0.035*	*− 6.691*	*0.000*	*− 0.310*	*− 0.166*	*− 0.794*	*0.119*	*− 1.033*	*− 0.554*

Entries in italics indicate parameters that influence repair foci with statistical significance of *p*<0.05

This is also obvious by the more shallow slope of the DSB foci induction in MSCs with higher age (Fig. 1b).

Pulsed-field gel electrophoresis of genomic DNA from irradiated MSCs of different age confirmed that the degree of radiation-induced DNA fragmentation by DSBs does not differ between young and old cells, despite the differences in the γH2AX/53BP1 foci frequencies (Additional file 3: Figure S2). This suggests that in vitro aging of MSCs leads to impairment of the DNA damage recognition without changing the susceptibility of the nuclear DNA for radiation-induced breakage.

### In vitro aging affects ATM-dependent, but not DNA-PK-dependent DNA foci

**Fig. 2:**
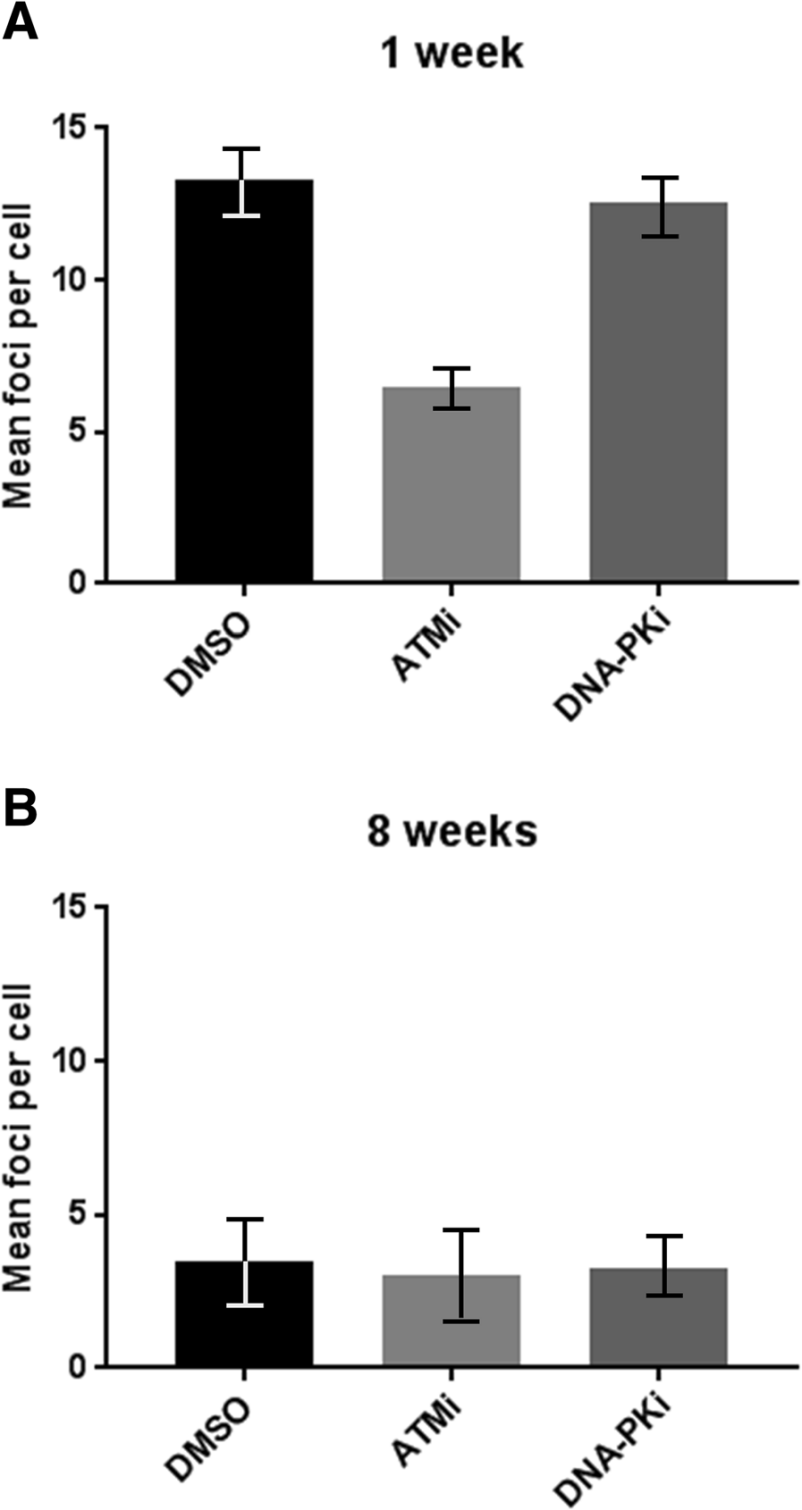
Effect of inhibition of ATM and DNA-PK on γH2AX- and 53BP1-foci formation in aging MSCs after exposure to irradiation. **a** One-week and **b** 8-week cultured MSCs were pre-incubated with DMSO and a chemical ATM (ATMi) or DNA-PK (DNA-PKi) inhibitor and irradiated with 6 Gy before γH2AX- and 53BP1-foci formation was detected 90 min post irradiation. Foci formation in at least 50 nuclei was automatically quantified using Keyence BZ-II Analyzer software, and the relative amount of repair foci was normalized to the 90 min value (mean values ± SEM, *n* = 3,)

Next, it was tested if the age-related change of DNA double-strand break recognition in irradiated MSCs can be attributed to either of the two major pathways of early DDR signaling. For this, young and old MSCs were incubated with chemical inhibitors for either ATM or DNA-PK, respectively, followed by 6 Gy γ-irradiation. DNA double-strand breaks as detected by γH2AX and 53BP1 foci after radiation exhibit a strong dependence on ATM signaling in 1-week-old MSCs (about 50% reduction, Fig. 2), whereas in 8-week-old cells, ATM inhibition had no measurable effect on damage recognition. In contrast to this, inhibition of the DNA-PK activity did not affect radiation-induced foci, neither in old nor in young cells. This suggests that the reduced DNA damage response in ex vivo aged MSCs is caused by a gradual loss of activity of the ATM-pathway.

### Kinetics of DNA double-strand break repair is slower in aging MSCs

**Fig. 3:**
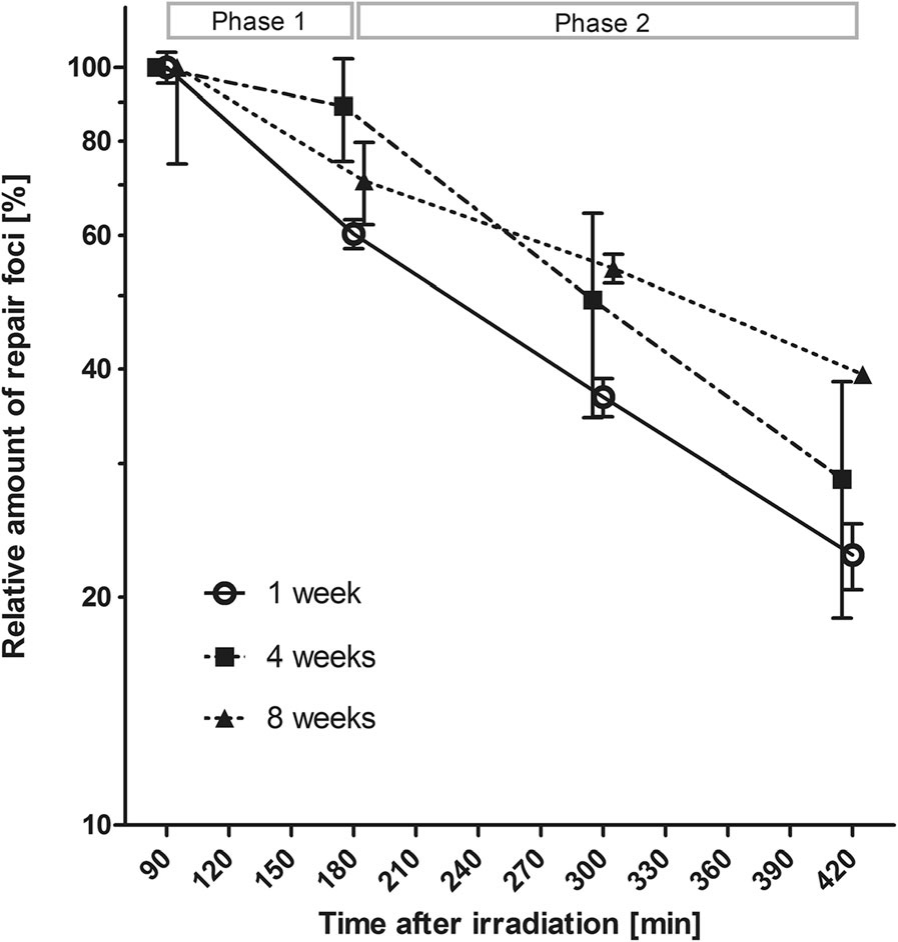
Kinetics of γH2AX- and 53BP1-foci formation in aging MSCs after exposure to 2 Gy. Aging MSCs were irradiated with 2 Gy before γH2AX- and 53BP1-foci formation was detected 90, 180, 300, and 420 min post irradiation. Foci formation in at least 50 nuclei was automatically quantified using Keyence BZ-II Analyzer software and the relative amount of repair foci was normalized to the 90 min value. Data plotted on a linear scale can be found in the Additional file 7: Figure S5. Mean repair half time in phase 1 and phase 2 and fraction of unrepaired DNA breaks is given in the Additional file 4: Table S1

To evaluate if DDR signaling would also affect the efficiency of DNA double-strand break repair, the progressive loss of γH2AX and 53BP1 foci of γ-irradiated young and old MSCs was determined. Following γ-irradiation, cells were incubated for different time intervals up to 7 h before immunofluorescence staining of the foci (Fig. 3). A single dose of 2 Gy was used to achieve a maximum sensitivity at later time points and to avoid pixel saturation at earlier time points. Clear specificity was observed in the repair kinetics between the 1-, 4-, and 8-week-old MSCs (Additional file 4: Table S1). Whereas in 1- and 4-week-old MSCs the majority of DNA breaks were repaired efficiently throughout the entire 7 h interval, the 8-week-old cells had biphasic kinetics with a fast initial phase and a slower second phase of DNA DSB repair as compared to the 1- and 4-week-old cells. The overall slower repair kinetics in older cells led to a significantly higher fraction of unrepaired DNA foci (39.3 ± 9.5%) in 8-week-old MSCs relative to the younger cells (22.9% or 28.6%, respectively, *p* < 0.05). Since unrepaired DNA damages can interfere with chromosomal segregation during mitosis and cause chromosomal instability in the progeny cells, it is important to evaluate if there are also more cytogenetic abnormalities following irradiation of in vitro aged MSCs.

### Higher frequency of spontaneous and radiation-induced micronuclei in aging MSCs

**Fig. 4:**
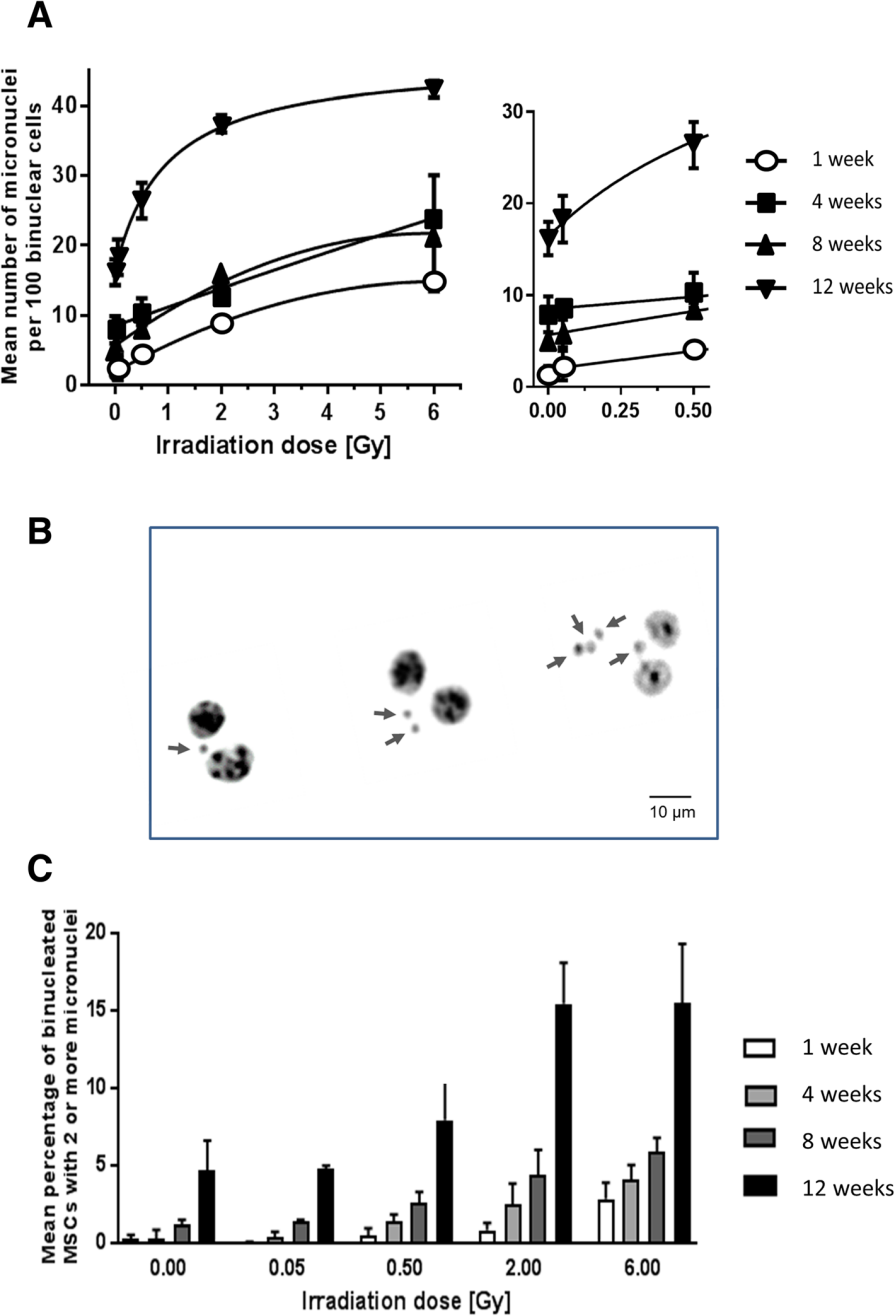
Frequency of micronuclei after exposure to irradiation in aging MSCs in vitro. **a** MSCs were cultured for 1, 4, 8, and 12 weeks under hypoxia in vitro. Mean number of micronuclei per 100 binuclear MSCs was detected after sham-irradiation (0 Gy) and 0.05, 0.5, 2, and 6 Gy irradiation and incubation for 5 days to allow repair of DNA damage in the presence of cytochalasin B. **b** Representative images of MSCs 5 days after 6 Gy γ-irradiation and DAPI staining showing cells with 1, 2, and 4 micronuclei. **c** Mean percentage of irradiated binucleated MSCs with two or more micronuclei (mean values ± SEM, *n* = 3)

To determine the impact of in vitro MSC expansion on chromosomal stability, the occurrence of micronuclei was evaluated after the first mitosis following γ-irradiation of young and aged MSCs. For this purpose, cells after 1, 4, 8, and 12 weeks in culture were irradiated and treated with cytochalasin B to arrest the cells at cytokinesis. This yields characteristic bi-nucleated cells and allows scoring for micronuclei at a defined stage after the mitosis (Fig. 4b). As expected, with increasing radiation dose significantly more micronuclei were scored in young (2 or 4 weeks in culture) as well as in older cells (8 and 12 weeks in culture), with a relative increase per Gy of 3–4 micronuclei. At each dose point, including 0 Gy, however, the frequency of micronuclei was the highest in 12-week-old MSCs, intermediate at 4- and 8-week-old MSCs and the lowest in 2-week-old MSCs (Fig. 4a). The influence both of cellular age and of radiation dose onto the number of micronuclei was highly significant (ANOVA test *p* < 0.0001). As shown by linear regression analysis, however, age in vitro of MSCs affected the occurrence of radiation-induced micronuclei only in an additive manner (Table 2). The radiation-response curves clearly indicate a parallel shift to higher values of micronuclei in aging MSCs, rather than a change in their slope (Fig. 4a).

**Table 2:** Multiple linear regression for radiation-induced micronuclei in aging MSCs. A dose-age interaction (*p* = 0.057) as suggested by ANOVA cannot be confirmed when in vitro age and radiation dose are evaluated by the strength of their individual effects and the compound effects. The majority of data in aged and irradiated MSCs suggest an additive interaction with age (constant shift to higher MN frequencies), rather than a sensitizing effect

	MN/cell	MN/cell	MN/cell	MN/cell	− 95%	95%	MN/cell	MN/cell	− 95%	95%
	Param.	St.Err.	*T*	*p*	Conf.Int.	Conf.Int.	Beta	St.Err. Beta	Conf.Int.	Conf.Int.
Const	0.0006	0.026	0.025	0.980	0.051	0.053				
Dose	*0.0266*	*0.009*	*2.907*	*0.005*	*0.008*	*0.045*	*0.384*	*0.132*	*0.119*	*0.649*
Age	*0.0188*	*0.004*	*5.038*	*0.000*	*0.011*	*0.026*	*0.472*	*0.094*	*0.284*	*0.660*
Dose × age	0.0026	0.001	1.946	0.057	0.0001	0.005	0.280	0.144	− 0.009	0.568

Entries in italics indicate parameters that influence micronuclei induction with statistical significance of *p*<0.05

This implies that in vitro aging can increase the frequency of cytogenetic alterations in MSCs by itself, rather than sensitizing the cells for the genotoxic effect of ionizing radiation.

### Clustering of multiple micronuclei in aging MSCs independent of radiation

To better understand the difference between radiation-induced and age-related micronuclei in MSCs, we analyzed the distribution of micronuclei in individual binucleated cells. The observed frequencies of cells containing single or multiple micronuclei were compared in the different experimental groups with the expected values assuming a Poisson distribution around the measured mean value (Additional file 5: Table S2, upper panel). For an easy comparison between the effects induced by in vitro aging and irradiation, two sets of data were selected that have similar mean values of micronuclei: 1-week-old MSCs after 6 Gy irradiation (0.215 ± 0.027 micronuclei/cell) and 12-week-old unirradiated MSCs (0.207 ± 0.041 micronuclei/cell). The frequency table shows that among the non-irradiated 12-week-old MSCs, there are more than expected cells with multiple micronuclei, but less cells having only one or no micronuclei, and about twice as many cells as expected with 2 or more micronuclei (Chi^2^ = 25.9, *p* < 0.0001). This is in contrast to “young” MSCs irradiated with 6 Gy of γ-rays, which have a similar mean number of micronuclei as old non-irradiated cells, but in which the distribution of events suggests a simple Poisson process. This was confirmed in all other datasets, showing that micronuclei were evenly distributed in MSCs after 1- or 4-week in vitro expansion, whereas in cells after 8- to 12-week in vitro expansion they occurred over-proportionally clustered in a few cells, independent on the radiation dose (Additional file 5: Table S2, lower panel). These data suggest that in aged MSCs, a fraction cells accumulate several DSBs and fail to repair them.

## Discussion

Like all types of adult stem cells, MSCs serve as a life-long reservoir for the generation of functional somatic cells and for the replacement of lost or functionally depleted cells. To ensure tissue homeostasis, MSCs have to keep their long-term proliferation potential and their multi-lineage differentiation capacity and protect their genome from mutations and structural chromosomal defects [32, 33]. Therefore, adult stem cells have an exceptionally high requirement to repair both replication-associated DNA damages as well as those induced by exogenous genotoxic factors.

Several studies investigated the influence of long-term ex vivo expansion of MSCs, but have largely focused on the cellular marker expression, on the growth rate, and on their multi-lineage differentiation potential [21, 34, 35]. It has been found that MSCs in vitro can be affected in their stem cell potency, telomeric length, and their growth potential. This is in contrast to embryonic stem cells which during in vitro culture appear to be relatively stable in terms of their stem cell potential and gene expression profile [31, 36]. Since genomic maintenance is an essential mechanism to prevent cells from accumulating cytogenetic changes and irreversible alterations in their DNA, we investigated the interaction of cellular aging during ex vivo expansion of MSCs and their DNA damage response after ionizing irradiation. Ionizing radiation, in the form of gamma- or X-rays or heavy accelerated subnuclear particles, is an unavoidable component of our natural environment and of our developed society. It can be estimated that in cells with an extremely slow turn-over like adult stem cells, the natural background radiation leads to a cumulative dose of several hundred mGy during an average human life of 75–80 years [36]. This cumulative dose is well in the range studied in this paper, and the question must be if the genome maintenance mechanisms in long-living mesenchymal stem cells can cope with this persistent challenge. An additional exposure with usually higher acute doses may result from various medical applications. Radiotherapy, nuclear medicine, and radiological imaging are frequently the method of choice to deal with malignant or acute diseases. Out-of-focus doses in tumor-directed radiotherapy with gamma- or orthovoltage X-rays can easily reach several Gy for large parts of the organism. These doses can induce a large number of DNA double-strand breaks in every cell, including in the small subset of adult stem cells. The first response of cells to these DNA breaks is the formation of DNA repair foci, an indication of proper damage recognition.

We have found in this study, however, that MSCs passaged over 8 weeks gradually lost their capacity to form γH2AX/53BP1 DNA foci. Whereas the background level of such foci in unirradiated cells does not vary over a period of 8-week in vitro aging, the frequency of induced foci following γ-irradiation was strongly dependent on age. The lower number of radiation-induced foci in 4- and 8-week-old relative to 2-week-old MSCs was not caused by a smaller number of DNA double-strand breaks, as shown by their similar level of DNA fragmentation in pulsed-field electrophoresis. This indicates that with longer expansion time ex vivo, MSCs do not per se accumulate less DNA damage, but that the recognition of radiogenic DNA damages is less efficient.

This might be the reason for the impaired processing of the DNA repair foci. When we measured the kinetics of DNA DSB repair in MSCs following 2 Gy of radiation, a reduction of repair kinetics in aged cells became evident (Additional file 7: Figure S5). As a consequence of this slower repair kinetics, older cells were left with a relatively higher number of residual breaks after completion of repair.

γH2AX foci form rapidly after DSB formation and have a key function in the repair of these lesions in mammalian cells, either by non-homologous end joining (NHEJ) or by homologous recombination (HRR) [37]. It has been shown that H2AX^−/−^ mice are radiosensitive, have a defect in recruiting important DNA repair proteins to sites of chromosomal breaks, and are deficient in V(D)J recombination [38]. Phosphorylation of H2AX is also crucial to preserve the chromatin integrity at sites of DNA double-strand breaks and to inhibit illegitimate repair pathways which could otherwise promote genetic instability [39]. It is therefore reasonable to conclude that reduced formation of γH2AX foci at a fixed level of DNA double-strand breaks increases the risk for genetic instability or DNA misrepair.

It was already shown by others that in vitro expansion of human MSCs involves the downregulation of DNA repair genes during senescence [34]. Interestingly, a reduced DNA double-strand break repair and an increased radiosensitivity were also found in human MSCs after their in vitro transformation by ectopic oncogene expression [40]. This underpins the importance of an efficient genome surveillance mechanism in MSCs after genotoxic stress and its role for the prevention of malignant transformation.

One of the earliest and most central elements of the DNA damage signaling is the phosphatidylinositol 3-kinase ATM [41]. Using a chemical inhibitor to test the involvement of this pathway, we found a reduction of ATM-dependent foci with increasing age in vitro, implying that ATM signaling is impaired during prolonged ex vivo expansion. Another PI3-kinase that can phosphorylate H2AX histones is DNA-PK. We found that the formation of radiation-induced DNA double-strand break foci in MSCs is independent on DNA-PK, both in young and in old cells. Although ATM and DNA-PK can both phosphorylate H2AX at sites of DNA double-strand breaks [42], they differ in their tissue specificity and expression of mutant phenotype. Whereas ATM germline mutations or deficiencies causes predisposition for malignancies [43, 44], neurological disorders [45], growth retardation, genetic instability in solid tissues [46], and accelerated aging after genotoxic stress [47], DNA-PK deficiencies are more limited in their phenotypic expression to the immune-system and gametogenesis [48]. One can therefore conclude that the age-related reduction of γH2AX/53BP1 foci in MSCs after γ-irradiation is caused by an impaired DNA damage response, presumably by regulatory changes in the ATM activity, rather than by changes in the histone H2AX target.

Despite differences in cell cycle and cell type specificity of the ATM and DNA-PK DDR signaling pathways, both are involved in the recovery from structural defects in the chromosomal DNA and therefore are important for normal segregation of sister chromatids to the daughter cells after mitosis. A general late effect of impaired DNA DSB repair is the occurrence of cytogenetic aberrations at or after cell division. Together with the age-related impairment of radiation-induced repair foci, we also found differences between young and old MSCs for a marker of cytogenetic damage, namely the occurrence of micronuclei. Whereas the frequency of DNA repair foci at a fixed radiation dose was much higher than the number of micronuclei per cell (about 0.1 MN in a single young MSCs irradiated with 6 Gy, as compared with about 13 γH2AX/53BP1 repair foci), the relative increase of both endpoints over the dose range from 50 mGy to 6 Gy was very similar. The influence of in vitro cellular age on the frequency of micronuclei, however, was a mirror image to the observations on DNA foci. Whereas the latter were strongly reduced in aging cells, micronuclei steadily increased in MSCs with longer periods of ex vivo proliferation. Although radiation dose and the extended time in vitro both had a promoting effect on the number of micronuclei, multiple regression analysis shows that the two interact only in an additive manner. Analysis of the distribution of micronucleus multiplicity in individual cells revealed a distinct picture, though showing that aging in vitro causes more clustering of these cytogenetic abnormalities in a subset of cells, whereas micronuclei induced by irradiation occurred more uniformly spread over all cells.

This suggests that despite the absence of a sensitizing effect of in vitro cellular age for the total number of radiation-induced micronuclei, a promoting effect can be assumed for their clustering in a few heavily affected cells. Considering that the malignant transformation is a multistep process starting from a few cells only, the occurrence of a rare cellular event by a factor of two or more can be an important risk factor, even if the absolute frequency of such events is low.

In this regard, it is interesting that recent studies also found evidence for an active role of micronuclei in the occurrence of chromothripsis [49, 50], a mechanism of single chromosome destabilization found in some malignancies [51].

## Conclusions

Taken together, we can conclude that with increasing time of in vitro expansion MSCs, an impaired recognition of radiation-induced DNA damage is evident. The increased number of micronuclei, however, is the result of acute radiation-induced abnormalities and the accumulation of endogenous damages over several cell generations. We also hypothesize that the instantaneous genotoxic effect of ionizing radiation and the more chronic cellular effect of in vitro aging both impair chromosomal stability, but following different mechanisms.

## Additional files

Additional file 1: **Figure S3.** Representative images of primary murine mesenchymal stem cells (MSCs) cultured for 1 week, 4 weeks, 8 weeks, and 12 weeks in vitro under hypoxic conditions passaged once per week. (TIF 3355 kb)

Additional file 2: **Figure S4.** Induced and spontaneous differentiation of mesenchymal stem cells after in vitro cultivation for (A) 1 week or (B) 12 weeks. (TIF 7446 kb)

Additional file 3: **Figure S2.** Detection of initial DNA double-strand breaks by pulsed-field electrophoresis. (TIF 1366 kb)

Additional file 4: **Table S1:** Half time of DNA double-strand break repair in first and second phase, % DNA double-strand breaks repaired in first and second phase and % of residual, unrepaired DNA double-strand breaks in MSCs of different in-vitro age. (TIF 126 kb)

Additional file 5: **Table S2:** Dispersion analysis of clustered micronuclei in individual cells. Observed frequencies of cells with multiple micronuclei are compared with expected frequencies assuming poisson distribution around the mena value. Upper panel: Comparison between 1 week old cells irradiated with 6 Gy and unirradiated 12 week old cells. Lower panel: Comparison between observed and expected frequencies of multiple micronuclei and cells of different in-vitro ages and after different radiation doses. (TIF 258 kb)

Additional file 6: **Figure S1.** Detection and distribution of γH2AX and 53BP1 foci in aging MSCs. (TIF 1180 kb)

Additional file 7: **Figure S5.** Kinetic if γH2AX- and 53B1-foci formation in aging MSCs after exposure to 2 Gy. (TIF 308 kb)

## Data Availability

The datasets and image files analyzed during the current study are available from the corresponding author on reasonable request.

## References

[1] Dennis JE, Merriam A, Awadallah A, Yoo JU, Johnstone B, Caplan AI (1999). A quadripotential mesenchymal progenitor cell isolated from the marrow of an adult mouse. J Bone Miner Res.

[2] Scharstuhl A, Schewe B, Benz K, Gaissmaier C, Buhring HJ, Stoop R (2007). Chondrogenic potential of human adult mesenchymal stem cells is independent of age or osteoarthritis etiology. Stem Cells.

[3] Stappenbeck TS, Miyoshi H (2009). The role of stromal stem cells in tissue regeneration and wound repair. Science (New York, NY).

[4] Squillaro T, Peluso G, Galderisi U (2016). Clinical trials with mesenchymal stem cells: an update. Cell Transplant.

[5] Krampera M, Pizzolo G, Aprili G, Franchini M (2006). Mesenchymal stem cells for bone, cartilage, tendon and skeletal muscle repair. Bone..

[6] Rohban R, Pieber TR (2017). Mesenchymal stem and progenitor cells in regeneration: tissue specificity and regenerative potential. Stem Cells Int.

[7] Awad HA, Butler DL, Boivin GP, Smith FN, Malaviya P, Huibregtse B (1999). Autologous mesenchymal stem cell-mediated repair of tendon. Tissue Eng.

[8] Paquet-Fifield S, Schluter H, Li A, Aitken T, Gangatirkar P, Blashki D (2009). A role for pericytes as microenvironmental regulators of human skin tissue regeneration. J Clin Invest.

[9] Melo FR, Bressan RB, Forner S, Martini AC, Rode M, Delben PB (2017). Transplantation of human skin-derived mesenchymal stromal cells improves locomotor recovery after spinal cord injury in rats. Cell Mol Neurobiol.

[10] Roubelakis MG, Trohatou O, Roubelakis A, Mili E, Kalaitzopoulos I, Papazoglou G (2014). Platelet-rich plasma (PRP) promotes fetal mesenchymal stem/stromal cell migration and wound healing process. Stem Cell Rev.

[11] Agay D, Scherthan H, Forcheron F, Grenier N, Herodin F, Meineke V (2010). Multipotent mesenchymal stem cell grafting to treat cutaneous radiation syndrome: development of a new minipig model. Exp Hematol.

[12] Abumaree M, Al Jumah M, Pace RA, Kalionis B (2012). Immunosuppressive properties of mesenchymal stem cells. Stem Cell Rev.

[13] Kallekleiv M, Larun L, Bruserud O, Hatfield KJ (2016). Co-transplantation of multipotent mesenchymal stromal cells in allogeneic hematopoietic stem cell transplantation: a systematic review and meta-analysis. Cytotherapy..

[14] Sage EK, Thakrar RM, Janes SM (2016). Genetically modified mesenchymal stromal cells in cancer therapy. Cytotherapy..

[15] Hagenhoff A, Bruns CJ, Zhao Y, von Luttichau I, Niess H, Spitzweg C (2016). Harnessing mesenchymal stem cell homing as an anticancer therapy. Expert Opin Biol Ther.

[16] Trounson A, McDonald C (2015). Stem cell therapies in clinical trials: progress and challenges. Cell Stem Cell.

[17] Franzen J, Zirkel A, Blake J, Rath B, Benes V, Papantonis A (2017). Senescence-associated DNA methylation is stochastically acquired in subpopulations of mesenchymal stem cells. Aging Cell.

[18] de Almeida DC, Ferreira MR, Franzen J, Weidner CI, Frobel J, Zenke M (2016). Epigenetic classification of human mesenchymal stromal cells. Stem Cell Reports.

[19] Tolar J, Nauta AJ, Osborn MJ, Panoskaltsis Mortari A, McElmurry RT, Bell S (2007). Sarcoma derived from cultured mesenchymal stem cells. Stem Cells.

[20] Miura M, Miura Y, Padilla-Nash HM, Molinolo AA, Fu B, Patel V (2006). Accumulated chromosomal instability in murine bone marrow mesenchymal stem cells leads to malignant transformation. Stem Cells.

[21] Wang Y, Zhang Z, Chi Y, Zhang Q, Xu F, Yang Z (2013). Long-term cultured mesenchymal stem cells frequently develop genomic mutations but do not undergo malignant transformation. Cell Death Dis.

[22] Capelli C, Pedrini O, Cassina G, Spinelli O, Salmoiraghi S, Golay J (2014). Frequent occurrence of non-malignant genetic alterations in clinical grade mesenchymal stromal cells expanded for cell therapy protocols. Haematologica..

[23] Torsvik A, Rosland GV, Svendsen A, Molven A, Immervoll H, McCormack E (2010). Spontaneous malignant transformation of human mesenchymal stem cells reflects cross-contamination: putting the research field on track - letter. Cancer Res.

[24] Christensen R, Alsner J, Brandt Sorensen F, Dagnaes-Hansen F, Kolvraa S, Serakinci N (2008). Transformation of human mesenchymal stem cells in radiation carcinogenesis: long-term effect of ionizing radiation. Regen Med.

[25] Tang Q, Chen Q, Lai X, Liu S, Chen Y, Zheng Z (2013). Malignant transformation potentials of human umbilical cord mesenchymal stem cells both spontaneously and via 3-methycholanthrene induction. PLoS One.

[26] Rieger K, Marinets O, Fietz T, Korper S, Sommer D, Mucke C (2005). Mesenchymal stem cells remain of host origin even a long time after allogeneic peripheral blood stem cell or bone marrow transplantation. Exp Hematol.

[27] Nicolay NH, Liang Y, Lopez Perez R, Bostel T, Trinh T, Sisombath S (2015). Mesenchymal stem cells are resistant to carbon ion radiotherapy. Oncotarget..

[28] Chen MF, Lin CT, Chen WC, Yang CT, Chen CC, Liao SK (2006). The sensitivity of human mesenchymal stem cells to ionizing radiation. Int J Radiat Oncol Biol Phys.

[29] Oliver L, Hue E, Sery Q, Lafargue A, Pecqueur C, Paris F (2013). Differentiation-related response to DNA breaks in human mesenchymal stem cells. Stem Cells.

[30] Nicolay NH, Sommer E, Lopez R, Wirkner U, Trinh T, Sisombath S (2013). Mesenchymal stem cells retain their defining stem cell characteristics after exposure to ionizing radiation. Int J Radiat Oncol Biol Phys.

[31] Höfig Ines, Ingawale Yashodhara, Atkinson Michael J., Hertlein Heidi, Nelson Peter J., Rosemann Michael (2016). p53-Dependent Senescence in Mesenchymal Stem Cells under Chronic Normoxia Is Potentiated by Low-Doseγ-Irradiation. Stem Cells International.

[32] Sperka T, Wang J, Rudolph KL (2012). DNA damage checkpoints in stem cells, ageing and cancer. Nat Rev Mol Cell Biol.

[33] Signer RA, Morrison SJ (2013). Mechanisms that regulate stem cell aging and life span. Cell Stem Cell.

[34] Galderisi U, Helmbold H, Squillaro T, Alessio N, Komm N, Khadang B (2009). In vitro senescence of rat mesenchymal stem cells is accompanied by downregulation of stemness-related and DNA damage repair genes. Stem Cells Dev.

[35] Alt EU, Senst C, Murthy SN, Slakey DP, Dupin CL, Chaffin AE (2012). Aging alters tissue resident mesenchymal stem cell properties. Stem Cell Res.

[36] Rosemann M, Korogodina VL, Mothersill CE, Seymour CB (2016). Radiation-induced aging and genetic instability of mesenchymal stem cells: an issue for late health effects?. Genetics, evolution and radiation.

[37] Kinner A, Wu W, Staudt C, Iliakis G (2008). Gamma-H2AX in recognition and signaling of DNA double-strand breaks in the context of chromatin. Nucleic Acids Res.

[38] Celeste A, Petersen S, Romanienko PJ, Fernandez-Capetillo O, Chen HT, Sedelnikova OA (2002). Genomic instability in mice lacking histone H2AX. Science (New York, NY).

[39] Helmink BA, Tubbs AT, Dorsett Y, Bednarski JJ, Walker LM, Feng Z (2011). H2AX prevents CtIP-mediated DNA end resection and aberrant repair in G1-phase lymphocytes. Nature..

[40] Worku M, Fersht N, Martindale C, Funes JM, Short SC (2013). Sequential transformation of mesenchymal stem cells is associated with increased radiosensitivity and reduced DNA repair capacity. Radiat Res.

[41] Lee JH, Paull TT (2007). Activation and regulation of ATM kinase activity in response to DNA double-strand breaks. Oncogene..

[42] Stiff T, O'Driscoll M, Rief N, Iwabuchi K, Lobrich M, Jeggo PA (2004). ATM and DNA-PK function redundantly to phosphorylate H2AX after exposure to ionizing radiation. Cancer Res.

[43] Bay JO, Uhrhammer N, Pernin D, Presneau N, Tchirkov A, Vuillaume M (1999). High incidence of cancer in a family segregating a mutation of the ATM gene: possible role of ATM heterozygosity in cancer. Hum Mutat.

[44] Broeks A, Urbanus JH, Floore AN, Dahler EC, Klijn JG, Rutgers EJ (2000). ATM-heterozygous germline mutations contribute to breast cancer-susceptibility. Am J Hum Genet.

[45] Allen DM, van Praag H, Ray J, Weaver Z, Winrow CJ, Carter TA (2001). Ataxia telangiectasia mutated is essential during adult neurogenesis. Genes Dev.

[46] Elson A, Wang Y, Daugherty CJ, Morton CC, Zhou F, Campos-Torres J (1996). Pleiotropic defects in ataxia-telangiectasia protein-deficient mice. Proc Natl Acad Sci U S A.

[47] Barlow C, Eckhaus MA, Schaffer AA, Wynshaw-Boris A (1999). Atm haploinsufficiency results in increased sensitivity to sublethal doses of ionizing radiation in mice. Nat Genet.

[48] Taccioli GE, Amatucci AG, Beamish HJ, Gell D, Xiang XH, Torres Arzayus MI (1998). Targeted disruption of the catalytic subunit of the DNA-PK gene in mice confers severe combined immunodeficiency and radiosensitivity. Immunity..

[49] Terzoudi GI, Karakosta M, Pantelias A, Hatzi VI, Karachristou I, Pantelias G (2015). Stress induced by premature chromatin condensation triggers chromosome shattering and chromothripsis at DNA sites still replicating in micronuclei or multinucleate cells when primary nuclei enter mitosis. Mutat Res Genet Toxicol Environ Mutagen.

[50] Terradas M, Martin M, Genesca A (2016). Impaired nuclear functions in micronuclei results in genome instability and chromothripsis. Arch Toxicol.

[51] Rode A, Maass KK, Willmund KV, Lichter P, Ernst A (2016). Chromothripsis in cancer cells: an update. Int J Cancer.

