# Supplementary figures and images for: Long-term culture of mesenchymal stem cells impairs ATM-dependent recognition of DNA breaks and increases genetic instability
Source: Stem Cell Res Ther. 2019 Jul 29;10:218. doi: 10.1186/s13287-019-1334-6 (PMC6664790; doi:10.1186/s13287-019-1334-6)

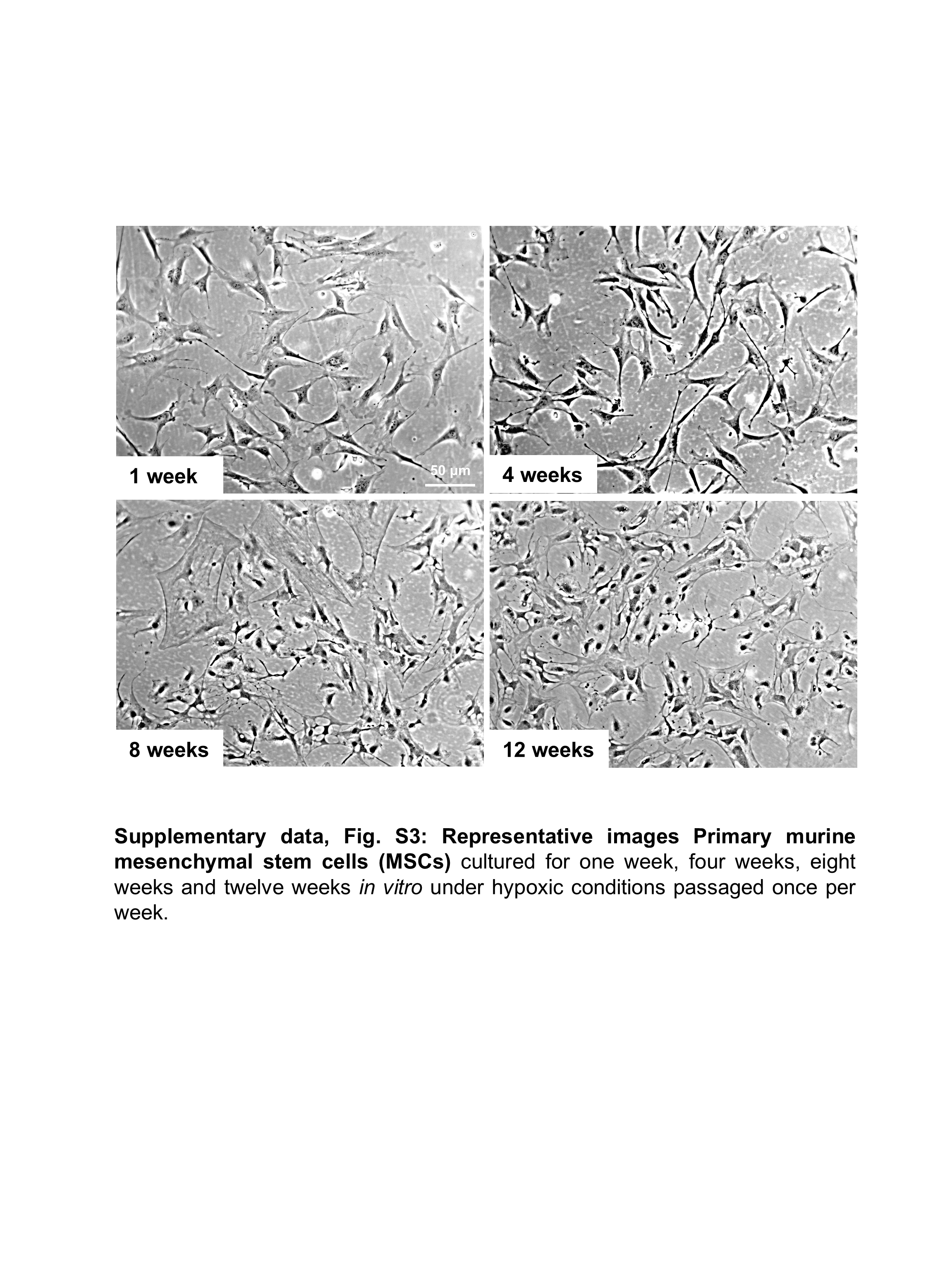

Supplement: Supplementary file 1 — Figure S3. Representative images of primary murine mesenchymal stem cells (MSCs) cultured for 1 week, 4 weeks, 8 weeks, and 12 weeks in vitro under hypoxic conditions passaged once per week. (TIF 3355 kb) [file 13287_2019_1334_MOESM1_ESM.tif]

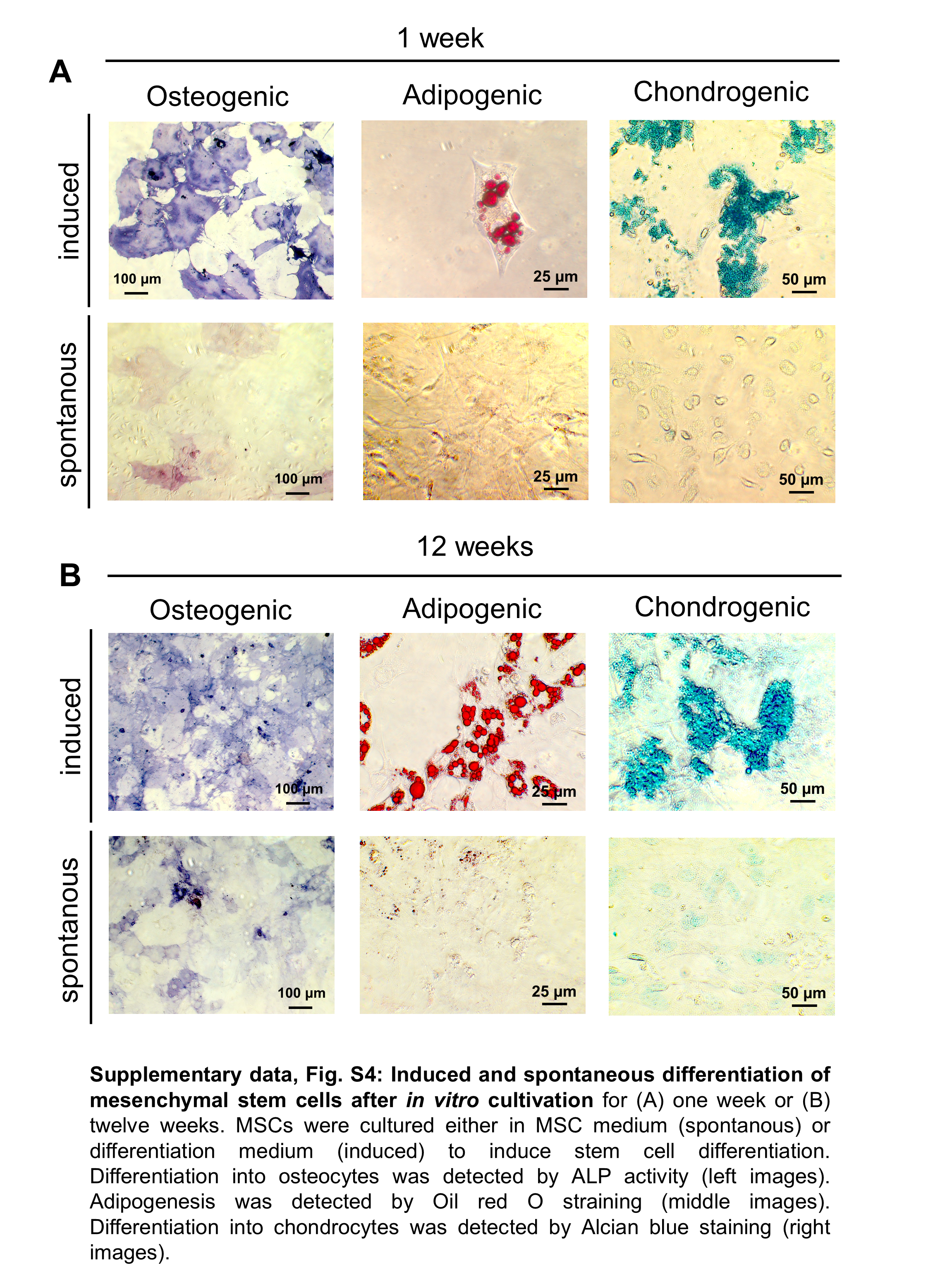

Supplement: Supplementary file 2 — Figure S4. Induced and spontaneous differentiation of mesenchymal stem cells after in vitro cultivation for (A) 1 week or (B) 12 weeks. (TIF 7446 kb) [file 13287_2019_1334_MOESM2_ESM.tif]

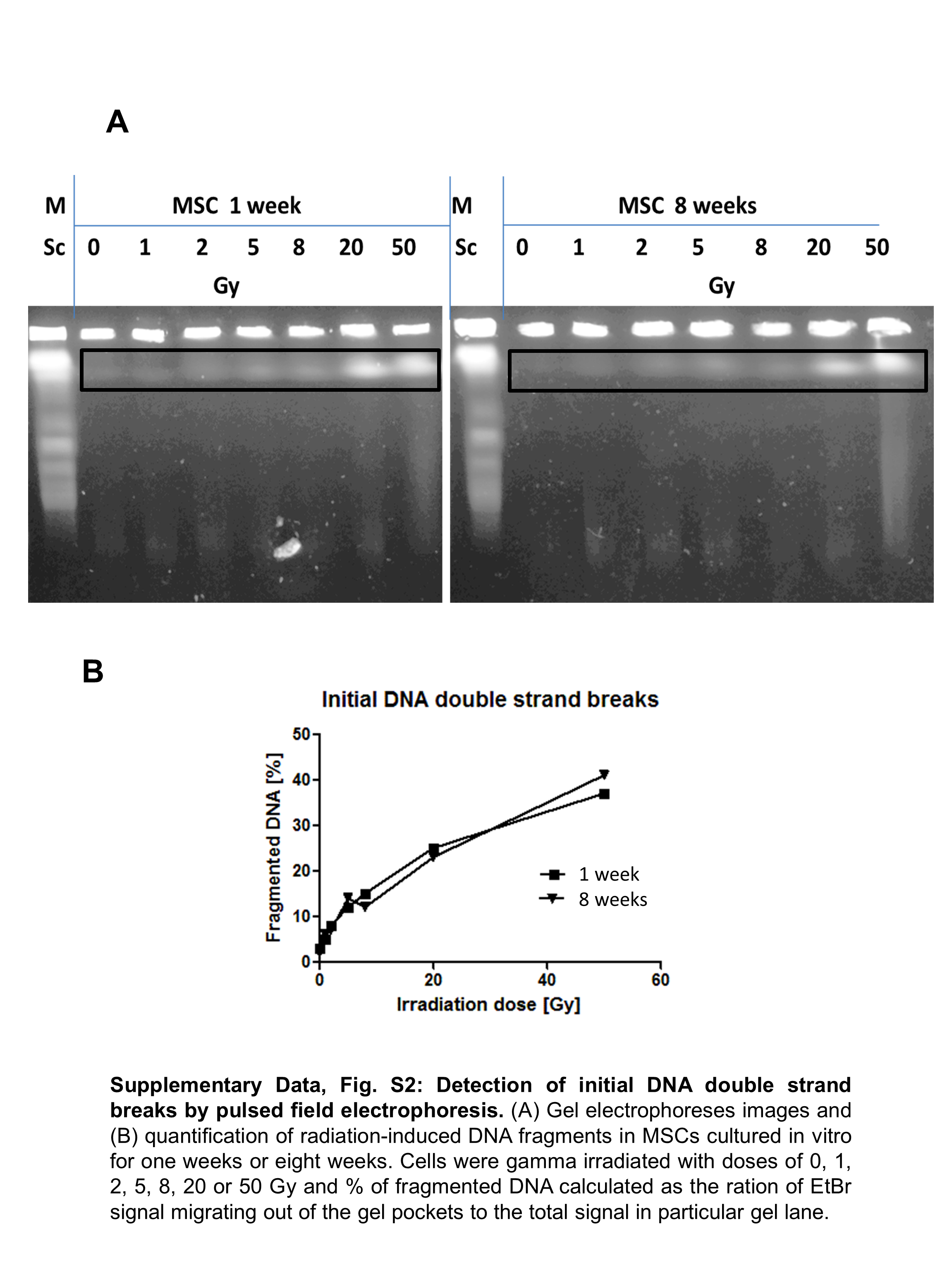

Supplement: Supplementary file 3 — Figure S2. Detection of initial DNA double-strand breaks by pulsed-field electrophoresis. (TIF 1366 kb) [file 13287_2019_1334_MOESM3_ESM.tif]

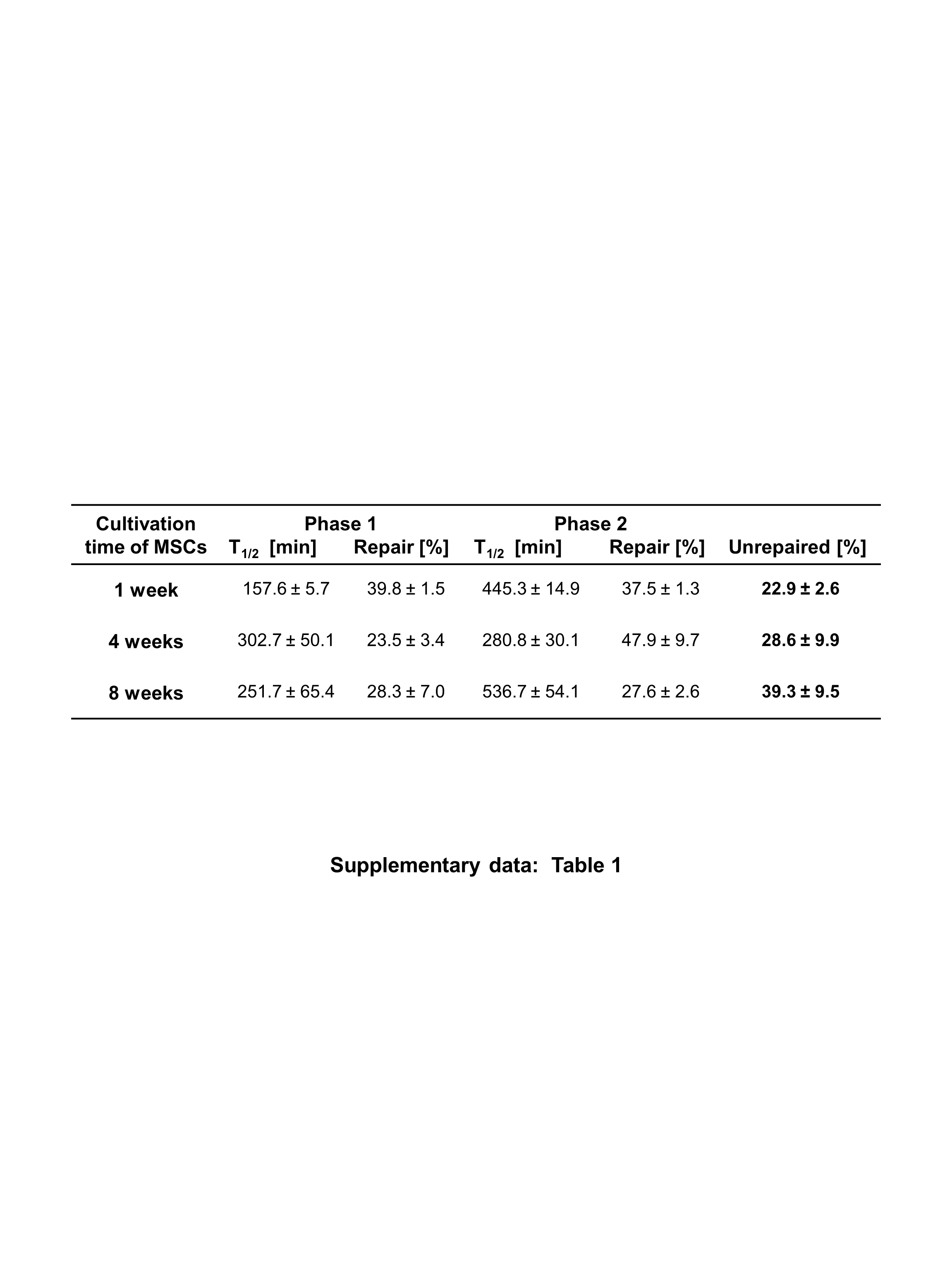

Supplement: Supplementary file 4 — Table S1: Half time of DNA double-strand break repair in first and second phase, % DNA double-strand breaks repaired in first and second phase and % of residual, unrepaired DNA double-strand breaks in MSCs of different in-vitro age. (TIF 126 kb) [file 13287_2019_1334_MOESM4_ESM.tif]

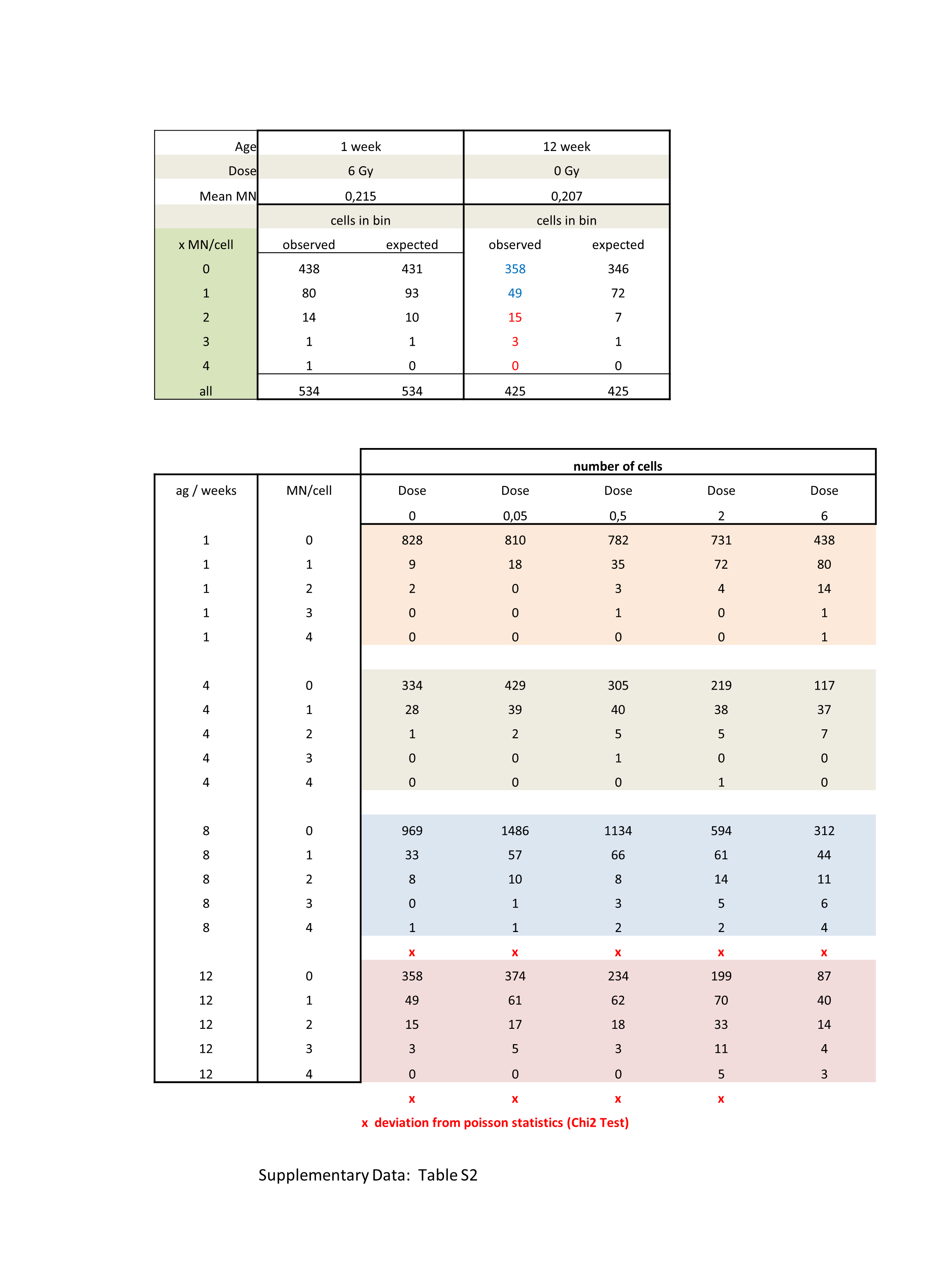

Supplement: Supplementary file 5 — Table S2: Dispersion analysis of clustered micronuclei in individual cells. Observed frequencies of cells with multiple micronuclei are compared with expected frequencies assuming poisson distribution around the mena value. Upper panel: Comparison between 1 week old cells irradiated with 6 Gy and unirradiated 12 week old cells. Lower panel: Comparison between observed and expected frequencies of multiple micronuclei and cells of different in-vitro ages and after different radiation doses. (TIF 258 kb) [file 13287_2019_1334_MOESM5_ESM.tif]

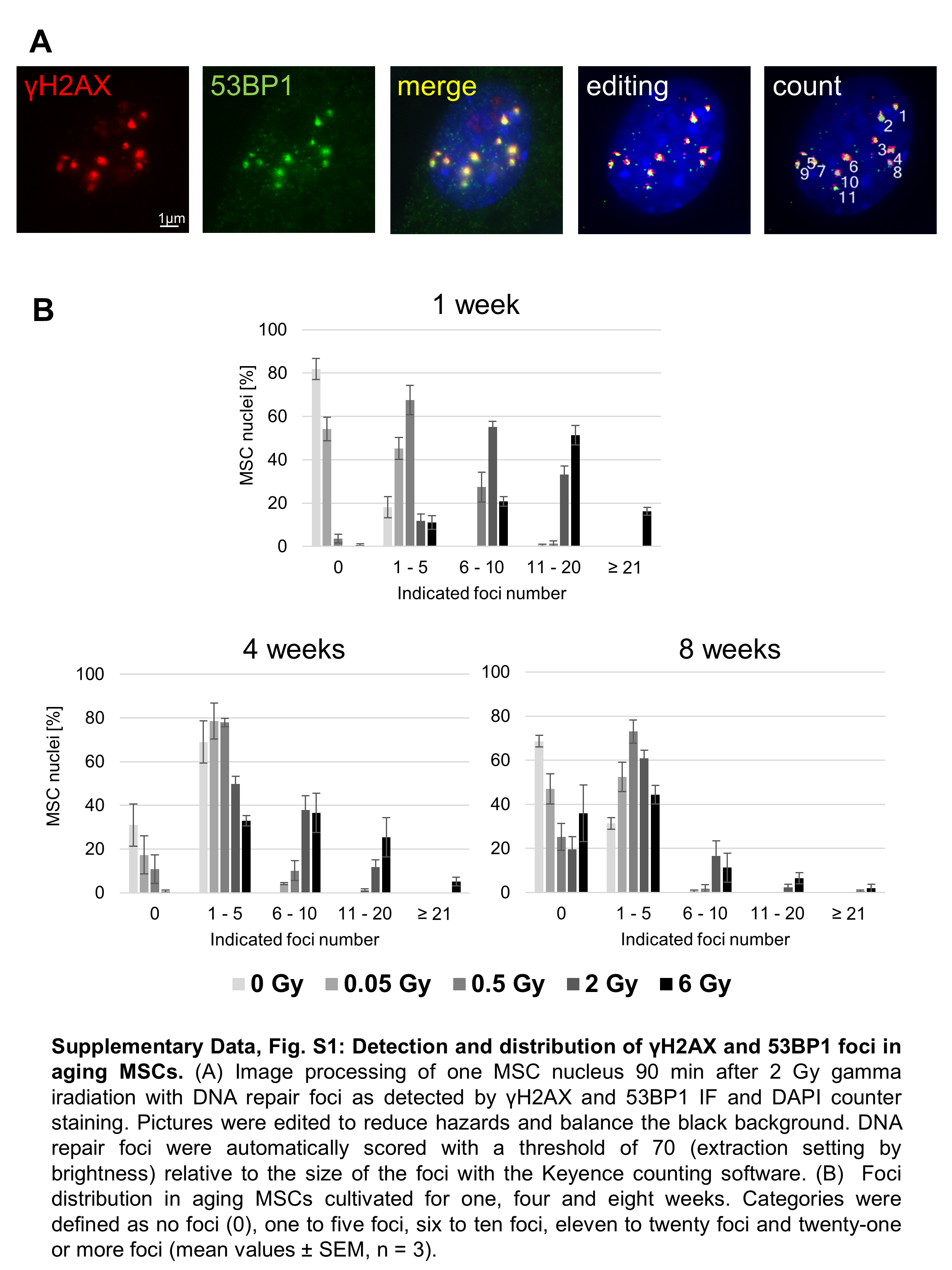

Supplement: Supplementary file 6 — Figure S1. Detection and distribution of γH2AX and 53BP1 foci in aging MSCs. (TIF 1180 kb) [file 13287_2019_1334_MOESM6_ESM.tif]

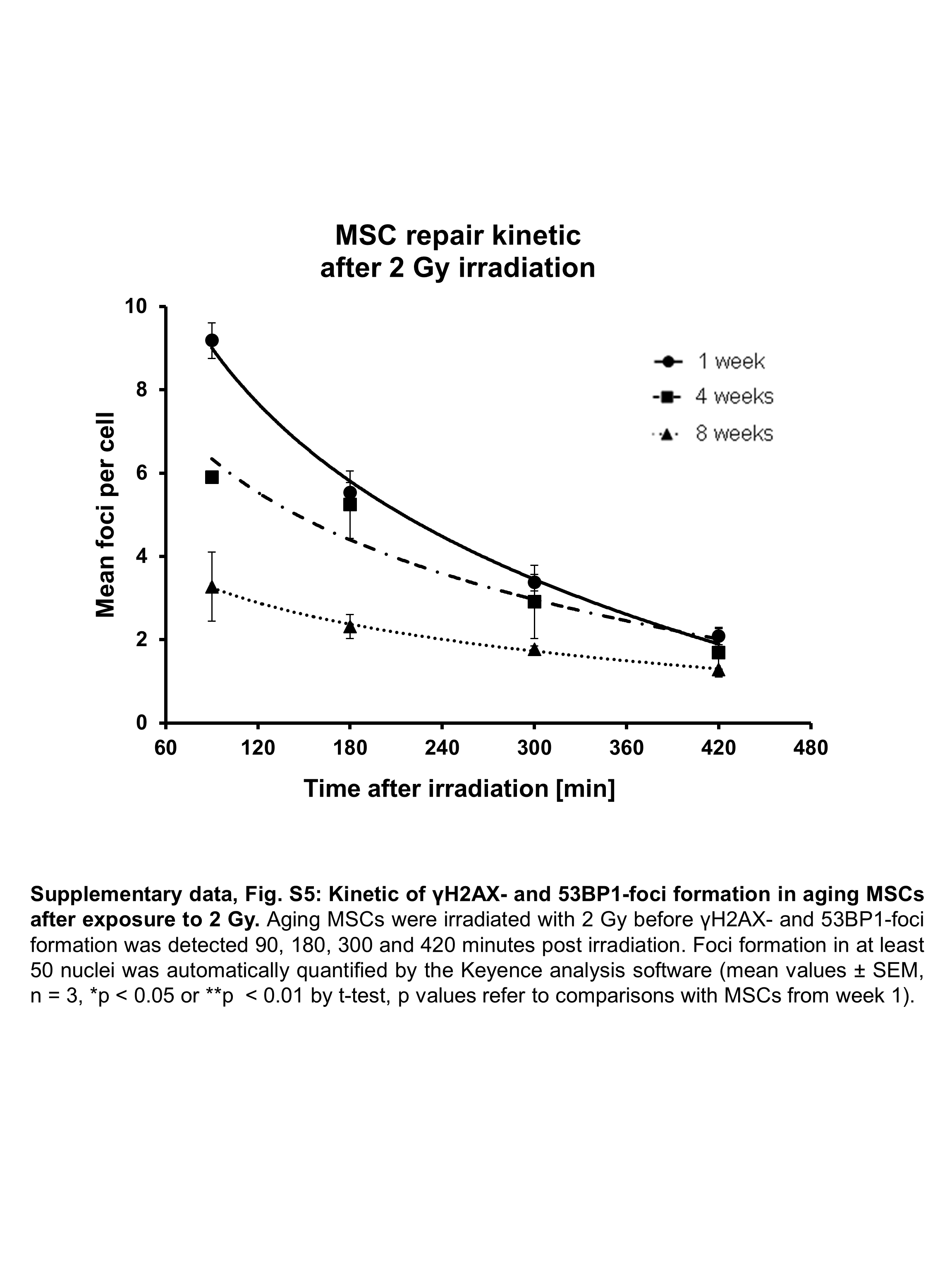

Supplement: Supplementary file 7 — Figure S5. Kinetic if γH2AX- and 53B1-foci formation in aging MSCs after exposure to 2 Gy. (TIF 308 kb) [file 13287_2019_1334_MOESM7_ESM.tif]
